# Maintaining mitochondria in beige adipose tissue

**DOI:** 10.1080/21623945.2019.1574194

**Published:** 2019-02-20

**Authors:** Xiaodan Lu

**Affiliations:** a Medical Diagnostic Research Center, Jilin Province People’s Hospital, Changchun, Jilin, China; b School of Medicine, Changchun University of Chinese Medicine, Changchun, Jilin, China

**Keywords:** Mitochondrial clearance, beige adipose tissue, Parkin, mitophagy

## Abstract

Individual cell types vary enormously in the amount of different organelles they contain. One such organelle is the mitochondrion. Understanding how mitochondrial levels are controlled is essential since so many disease states seem to involve mitochondrial function. The beige adipocyte is an inducible form of adipocyte that emerges in response to cold exposure and some other external stimuli. To perform its thermogenic function, its level of mitochondria increases dramatically. If the stimuli are removed the mitochondrial levels return to base line. Following the withdrawal of external stimuli, beige adipocytes directly acquire a white fat-like phenotype through mitophagy-mediated mitochondrial degradation. The beige cell is therefore a dynamic model for studying the mechanism of mitochondrial biogenesis and degradation.

## A dynamic model of mitochondrial biogenesis and degradation

Mitochondria misregulation occurs in human diseases, such as obesity, diabetes and cancer.^1^ The beige cell is a type of cold-inducible, mitochondrian-rich adipocyte and appears in the white adipose tissue (WAT).^2^ Adult human do not have interscapular brown adipose tissue (BAT), but showed brown-adipose-tissue activity in the supraclavicular region by static positron emission tomography of ^18^F-fluorodeoxyglucose in combination with computed tomography (^18^F-FDG PET-CT).^3,4^ The BAT activity is significantly related with cold-challenge, body mass index and age, which are actually beige adipocytes.^5,6^ It can be distinguished from the classical interscapular brown adipose tissue (iBAT). Classical brown adipocytes develop prenatally from a *Myf5, Pax7, Engrailed-1* positive progenitor population.^7–9^ Beige adipocytes postnatally develop from *PDGFα/β, Ebf2, Sca-1, SMA* positive progenitor population.^10-13^ Mitochondria biogenesis in white adipose tissue (WAT) can be activated by cold or β3 adrenergic agonist (β3-AR).^14^ In the past decade, research interests were intensely focused on the transcriptional control of those mitochondrial-rich adipocyte. Transcription factors *PRDM16, PGC-1α, et al*, switch on beige adipose specific gene transcription and protein expression, thus promote mitochondria biogenesis in WAT.^15,16^ Recently, a new type of glycolytic beige adipocyte is found from β-adrenergic receptor less mouse under chronic cold adaptation, in which presented enhanced glucose oxidation.^17^ The thermal stress induced progenitor cell plasticity in adipose tissue is still on the beginning of its discoveries.

Adipose tissue is able to sense environmental temperature and secretary factors.^18^ Growth factors are regulating fat storage and fatty acid transport. Beige cell can be induced by inhibiting several cellular growth factors signaling pathways such as those controlled by VEGF-A, VEGF-B, PDGFRα and TGFβ pathway.^19-21^ Exercise training results in adaptations of mitochondria biogenesis in both skeletal muscle and subcutaneous adipose.^22-24^ At the epigenetic level, methylation or acetylation enzymes are important in sensing and regulating the differentiation of beige cell. For example, *EHMT1* controls brown adipose cell fate and thermogenesis through the PRDM16 complex. Beige cell can be induced by the external β3-AR agonist CL316,243 in WAT of wild type mice, but not in the adipose specific *EMHT1* knockout mice.^25^ The Sirtuin family is a class of stress responsive protein deacetylase and mono-ADP ribosyltransferase enzymes. The Chang lab has discovered that Sirt1 is playing a major role in high-fat-diet induced liver metabolic damage.^26^ Cold-inducible Sirt6 regulates thermogenesis in both brown and beige fat.^27^

## Beige-to-white transition

The balance between energy-storage and energy-expenditure is always a systematic regulation process. When normal conditions are restored, the enriched mitochondria in beige cells disappear. After withdrawal of cold or β3-AR agonist stimuli, there always follows mitochondria degradation and the beige-to-white transition in WAT.^28^ The mitochondria uncoupling protein UCP1 and the oxidative phosphorylation complex OXPHOS are specific markers of mitochondria formation during the transition to beige adipocytes. As the Kajimura lab reported recently, the expression of mitochondria marker UCP1 and OXPHOS were both rapidly decreased in the beige adipocyte after withdrawing stimuli.^29^ During beige-to-white adipocyte transition, gene-annotation enrichment analysis found that the majority of the cluster I gene was related to mitochondria in cellular components, the electron transport chain and oxidation reduction related biological process.^14^ Thus, the phenotype of mitochondria degradation right after withdrawal of stimuli is well established in mouse inguinal adipose tissue.

The mitochondrial uncoupling protein UCP1 is an important marker for brown adipose tissue as well as beige adipose, and has been linked to thermogenesis. However beige fat thermogenesis is UCP1 dispensable. A recent study has discovered UCP1-independent thermogenesis pathways specifically in beige adipose. SERCA2b-mediated calcium cycling regulates UCP1-independent thermogenesis.^29^ Enhanced Ca2+ cycling by activation of α1 – and/or β3-adrenergic receptors or the SERCA2b-RyR2 pathway stimulates UCP1-independent thermogenesis in beige adipocytes. The beige adipocytes of UCP1 deficient mice presented enhanced glycolysis, tricarboxylic acid metabolism and pyruvate dehydrogenase activity for ATP-dependent thermogenesis. In *UCP1* knockout mice, beige cell can be highly induced by β3-AR agonist CL316,243, as well as mitochondrial degradation directly acquired after stimuli withdraw.^28^ The oxygen consumption rate (OCR) of WAT decreased to wild type OCR level after 15 days, which presented a UCP1-independent manner in the beige-to-white transition. Thus, beige cell biogenesis and degradation of mitochondria is UCP1-independent.

The majority of studies about mitochondrial integrity are in mice for which the role of housing temperature in determining the relevance of any outcomes should be considered. To remove the temperature induced differences, a comparison in ambient temperature and in thermoneutrality (30°C) could be set up respectively for the animal study of mitochondria clearance in beige adipose tissue.^28^

## Mitophagy controls mitochondrial quantity and quality

Mitophagy is the degradation of mitochondria by autophagy.^30^ To maintain the integrity and function of cells, it is important to eliminate damaged and aged mitochondria.^31-33^ During the beige-to-white transition, mitochondria are degraded in the adipose tissue by activation of autophagy.^14^ Transcriptional regulators of mitochondrial biogenesis *Pgc-1α, Nrf1/2, Tfam, et al*. directly decrease in the early phase of beige-to-white transition.^34-36^ Changes in the autophagy and lysosome pathways were highly relevant in the gene enrichment analysis. Based on some sets of gene profiling data, autophagy related genes *Atg5, Atg4b, Atg12, Atg16* were increased in the transition process.^37-40^ In others, autophagy related components and lysosomal enzyme related genes *Cts, Arsg, Naga* were also highly increased during the transition.^41-43^ The microtubule-associated protein 1A/1B-light chain 3 (LC3) is known to form a stable association with the membrane of autophagosomes.^44,45^ When *GFP-LC3* mice are employed in the experiments, GFP-LC3 and mitochondria marker Tom20 co-localization indicates autophagosome formation. After 7 days of CL316,243 treatment to induce the beige phenotype, the number of GFP-LC3 punctate was significantly decreased in the beige adipose. 15 days after stopping the treatment, the autophagic flux was back to a natural level. LC3-II protein expression was consistent with the co-localization result. The degradation of LC3-II has indicated that autophagy/ mitophagy process is attenuated by external β3-AR stimuli in beige cell.

Mitophagy plays a major role in beige cell mitochondria clearance. In a recent study, we employed the genetic model of *mt-Keima* mouse, which specifically expresses Keima protein in the mitochondria.^29^ The coral derived fluorescent protein Keima senses environmental pH value, giving green fluorescence in a regular cellular environment.^46,47^ In mitophagy, when the lysosome structure is formed and an acidic environment is generated, Keima protein gives a red fluorescence. The red to green fluorescence signal ratio can be precisely quantified by flow cytometry. The *mt-Keima* mouse presented a high level mitophagy-red/green ratio in white adipose tissue. After injecting mice for 7 days with the β3-AR agonist CL316,243 beige adipocyte biogenesis and attenuated mitophagy were induced in the inguinal adipose tissue and the red/green ratio shifted back to normal. After withdraw of the stimuli, the mitophagy signal gradually increases and recovers to regular level in 15–30 days. Thus using the *mt-Keima* mouse to measure the adipose tissue mitophagy stage or level reveals that the external β3-AR stimuli also induces beige cell biogenesis by attenuating the high levels of mitophagy that occur in normal WAT. Recently, Shirihai lab demonstrated that peridroplet mitochondria have higher pyruvate oxidation and ATP synthesis capacity, with unique structure and function that supports triacylglyceride synthesis.^48^ It opens up a new area in mitochondria biology. Meanwhile, the mitophagy levels between peridroplet mitochondria and cytoplasmic mitochondria in different environmental or physiological conditions could be characterized by the *mt-Keima* model.

PINK-Parkin signaling is the key regulator of mitochondrial degradation.^49^ Loss of mitochondrial membrane potential (depolarization) leads to PTEN-induced putative kinase 1 (PINK1) accumulation on the mitochondrial outer membrane.^50^ PINK1 recruits Parkin, an E3 ubiquitin-protein ligase, which ubiquitinates proteins on the outer mitochondrial membrane and starts the autophagic degradation of dysfunctional mitochondria. At a cellular level, PINK1-Parkin mediated mitophagy can be visualized by FCCP or CCCP treatment in the Parkin over-expressed model.^51^ CCCP is able to induce Parkin translocation to the mitochondria in mature beige adipocytes.^28^ Accumulation of damaged mitochondria has been linked to Parkinson’s disease, Alzheimer’s disease, diabetes and age-related disorders, but the mechanism of accumulated mitochondria clearance in adipose tissue is still not clear.^52-55^ In a study of Drosophila, Parkin mutant flies significantly decreased the rate of mitochondrial protein turnover.^56^ In the mouse adipose beige-to-white transition, Parkin also mediates the mitochondria clearance. After external β3-AR stimuli, beige cells can be induced in both wild type mice and Parkin deficient mice.^28^ 15 days after withdrawing the stimuli, beige cells disappeared in the wild type mouse, but still existed in the WAT of Parkin deficient mouse. When the oxygen consumption rate (OCR) was measured by Seahorse method the Parkin deficient mouse sustained a significant high level OCR than the wild type mouse in both basal and isoproterenol stimulated condition, which indicates that Parkin-dependent mitophagy is the key mechanism in beige cell mitochondrial clearance.

## Control mitochondrial clearance by phosphorylating parkin

**Figure 1.**
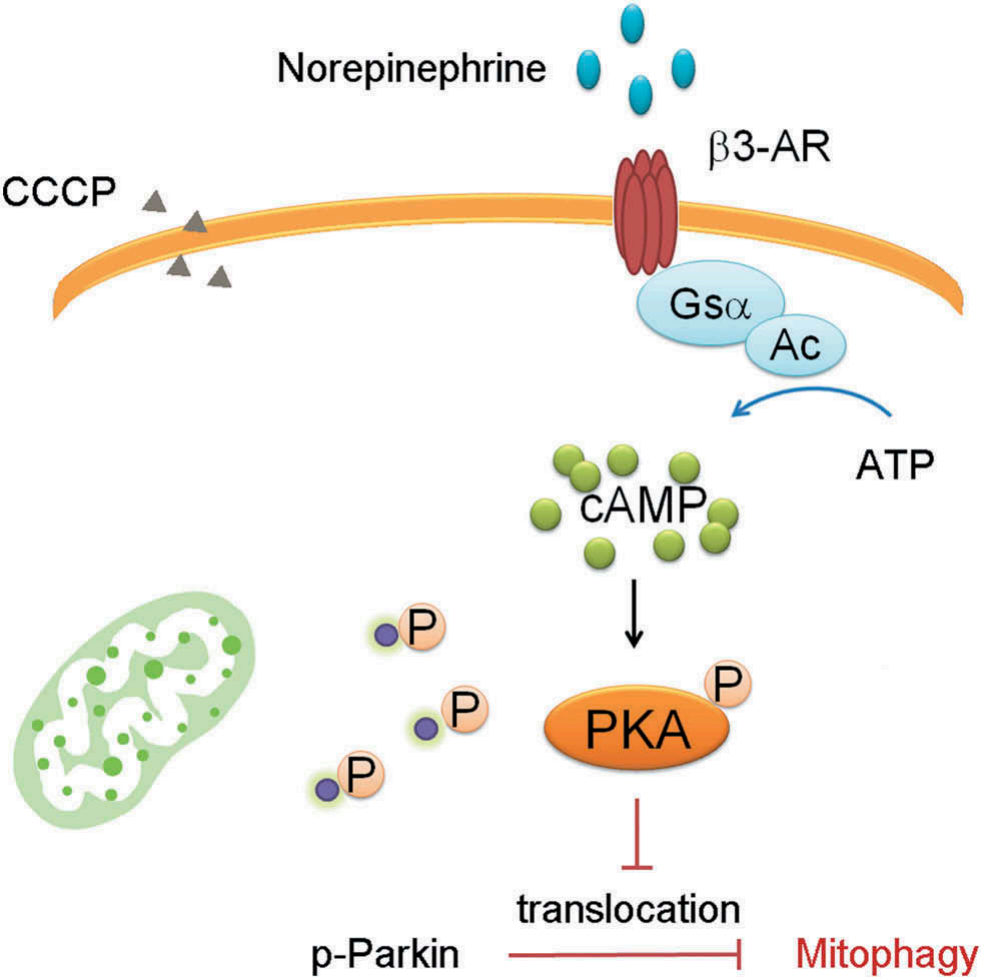
Parkin Phosphorylation attenuats mitophagy.

Parkin-mediated mitophagy plays a major role in beige cell mitochondrial clearance. In the CCCP induced-mitophagy beige cell model, Parkin translocation can directly be visualized. Norepinephrine can stimulate the cAMP signaling through β3-AR, activating PKA phosphorylation which phosphorylates downstream proteins.^6^ Parkin translocation can be attenuated by pre-treatment of norepinephrine stimulated β3-AR signaling, presumably inhibiting mitophagy.^28^ Pre-treatment with PKA inhibitors prevents the attenuated translocation when Parkin phosphorylation is preventing mitochondria degradation. In immunoprecipitation assay, norepinephrine induces Parkin phosphorylation which can also be blocked by the PKA inhibitors. The results suggest that Parkin recruitment into the mitochondria can be regulated by PKA signaling (Figure 1).

Recently, the Daumke and Wang labs have independently discovered that the structural plasticity of mitochondrial crista junctions is controlled by MIC60/Mitofilin.^57,58^ PINK1 phosphorylates inner mitochondrial membrane protein MIC60, which stabilizes MIC60 oligomerization. An earlier research was also focused on this pathway and found that PKA activation reduces PINK1 protein levels through phosphorylation of MIC60 and prevents the recruitment of Parkin to the mitochondria.^59^ Nevertheless, the function of MIC60/Mitofilin in beige cell mitochondria clearance is still unclear. Temporal inhibition of the MIC60-Parkin axis in the adipose tissue could be a novel approach to retain thermogenic beige adipocytes.

## Conclusions

A powerful tool to understand how mitochondrial levels are controlled is the beige adipocyte, an inducible regulator of thermogenesis. In the white to beige transition, mitochondrial levels are increased. In the beige to white transition, they are lowered. In both cases, the change in mitochondrial level is due to both expression of mitochondrial genes and the control of mitophagy. The uncoupling marker UCP1 does not play an important role in this regulation. Using single cell measurements of RNA levels, a complete description of the control of mitochondrial levels in adipocytes should soon be available. Such data should provide insights into how mitochondrial levels are regulated and into metabolism associated human diseases.

## Ackowledgements

The author is grateful to S. Kajimura and R. B. Kelly (UCSF) for their scientific discussions.
